# Genetic Variation for Wild Populations of the Rare and Endangered Plant *Glyptostrobus pensilis* Based on Double-Digest Restriction Site-Associated DNA Sequencing

**DOI:** 10.3390/cimb47010012

**Published:** 2024-12-30

**Authors:** Yongrong Huang, Yu Li, Xiaojie Hong, Suzhen Luo, Dedan Cai, Xiangxi Xiao, Yunpeng Huang, Yushan Zheng

**Affiliations:** 1 College of Forestry, Fujian Agriculture and Forestry University, Fuzhou 350002, China; yongrong1224@126.com (Y.H.); yulitrees@163.com (Y.L.); fafuhxj@163.com (X.H.); 2 Fujian Academy of Forestry, Fuzhou 350012, China; xiaoxiangxi973@163.com (X.X.); hyp1234888@126.com (Y.H.); 3 Youxi State-Owned Forest Farm of Fujian Province, Youxi 365100, China; luozi7208@163.com; 4 Jianning State-Owned Forest Farm of Fujian Province, Jianning 354500, China; 15259821857@163.com

**Keywords:** *Glyptostrobus pensilis*, ddRAD-seq, genetic diversity, genetic structure, conservation

## Abstract

*Glyptostrobus pensilis* is an endangered tree species, and detecting its genetic diversity can reveal the mechanisms of endangerment, providing references for the conservation of genetic resources. Samples of 137 trees across seven populations within Fujian Province were collected and sequenced using double-digest restriction site-associated DNA (ddRAD-seq). A total of 3,687,189 single-nucleotide polymorphisms (SNPs) were identified, and 15,158 high-quality SNPs were obtained after filtering. The genetic diversity in the populations was found to be low (*H_o_* = 0.08630, *H_e_* = 0.03475, *π* = 0.07239), with a high genetic differentiation coefficient (*F_st_*). When *K* = 4, the coefficient of variation (CV) error value was minimized, suggesting that the 137 individuals could be divided into four groups, with frequent gene flow between them. Principal component analysis (PCA) divided the seven populations into two major categories based on their north–south geographic location. The clustering was consistent with those obtained from the PCA. The main reasons for the endangerment of *G. pensilis* are likely to be poor natural regeneration, human disturbances, and climatic factors. It is recommended that methods such as in situ conservation, ex situ conservation, and the establishment of germplasm banks be implemented to maintain the genetic diversity of *G. pensilis* populations.

## 1. Introduction

*Glyptostrobus pensilis*, belonging to the Cupressaceae family, is a semi-evergreen tree that thrives in light and moisture-tolerant conditions. It is a unique monotypic relict species native to China, and a critically endangered wild plant under first-class state protection. This species is primarily distributed in the Fujian, Guangdong, Jiangxi, Guangxi, Yunnan, and Hunan Provinces of China, as well as Central Vietnam and Eastern Laos [1,2], growing mostly in neutral or slightly alkaline alluvial soils. These trees are sporadically found at altitudes ranging from 100 to 1300 m in paddy fields, marshes, and alpine meadows. According to the second national survey of protected wild plant resources, the population of *G. pensilis* in Fujian Province accounts for more than half of the national total [3,4].

*G. pensilis* serves numerous functions. Its lightweight root wood is used for lifesaving devices and corks. Its bark scales can be extracted to obtain tannin and used in tanning fishing nets. Its branches, leaves, and fruits have certain medicinal values, and its sturdy trunk and well-developed root system make it not only an ornamental species but also an effective solution for windbreaking and bank stabilization [5]. However, the survival of *G. pensilis* faces severe challenges due to habitat reduction and human disturbances. Firstly, the natural regeneration capacity under mother trees is poor [6], and it is sensitive to climatic conditions, with strict habitat requirements affecting its continuous propagation, leading to an uneven population age structure. Secondly, human activities are contributing to the fragmentation of habitats, and increasing competition for resources from other tree species and habitats has led to a sharp decline in its numbers [7]. Therefore, measures for the further protection and restoration of *G. pensilis* populations are urgently needed.

Genetic diversity is the basis for species to adapt to environmental changes and survive in the long term, and detecting and assessing genetic diversity will help to formulate more effective conservation strategies. Fagen et al. [8] conducted a study on the genetic structure and diversity of *G. pensilis*, revealing low genetic diversity and significant genetic differentiation both within and among populations, providing a theoretical basis for its conservation and development. Wu et al. [9] used inter-simple sequence repeat (ISSR) primers to analyze 72 individuals, obtaining similar results, namely low genetic diversity. Le et al. [10] studied the population genetic structure and diversity of *Sonneratia caseolaris* using ISSR molecular markers, providing new perspectives on the genetic resource conservation and development of that species. Kanna et al. [11] studied the genetic diversity of *Terminalia bellerica* to assess the growth and physiochemical characteristics of different populations. Danusevičius et al. [12] studied the genetic variability structure within and among populations of Scots pine, finding that the intra-population genetic variability structure was higher than the inter-population genetic variability structure, revealing greater genetic diversity within northern conifer species populations. Iwona et al. [13] identified the origins and key populations of black locust through a study of genetic diversity. The genetic variation within species is particularly important for the introduction of measures for the conservation of genetic resources, as well as the execution of breeding programs.

Reduced representation genome sequencing technology uses restriction enzymes to digest genomic DNA, selecting certain regions for high-throughput sequencing, thereby developing many genetic markers using partial genomic sequences to represent the whole genomic sequence [14,15]. Following a period of development, the current simplified genome sequencing methods mainly include restriction site-associated DNA sequencing (RAD-seq), genotyping by sequencing, and specific locus-amplified fragment sequencing. Among them, RAD-seq includes single-digest RAD sequencing, double-digest restriction site-associated DNA sequencing (ddRAD-seq), and type IIB digest RAD sequencing. RAD-seq allows for the acquisition of numerous genetic markers from non-reference genomes, with lower sequencing costs and simpler data processing, making it particularly suitable for large-scale genetic diversity and population structure analysis [16]. It is now widely used in various fields, such as molecular marker development, genetic mapping, and analyses regarding the genetics of wild populations [17].

This study aims to analyze the genetic variation of *G. pensilis* populations using ddRAD-seq, to reveal the characteristics of the populations from different geographical locations, to assess the levels of genetic diversity and structure, and to further explore the endangerment mechanisms, providing a basis for the conservation and utilization of genetic resources

## 2. Materials and Methods

### 2.1. Materials

**Figure 1:**
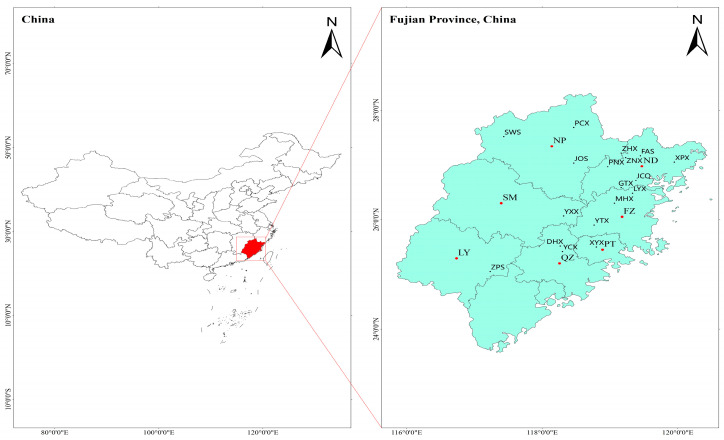
Sampling locations of seven populations of *Glyptostrobus pensilis* in Fujian Province. ND: Ningde; NP: Nanping; FZ: Fuzhou; SM: Sanming; PT: Putian; QZ: Quanzhou; LY: Longyan; XPX: Xiapuxian; FAS: Fu’anshi; ZNX: Zhouningxian; JCQ: Jiaochengqu; PNX: Pingnanxian; GTX: Gutianxian; PCX: Puchengxian; ZHX: Zhenghexian; JOS: Jian’oushi; SWS: Shaowuxian; LYX: Luoyuanxian; MHX: Minhouxian; YTX: Yongtaixian; XYX: Xianyouxian; DHX: Dehuaxian; YCX: Yongchunxian; ZPS: Zhangpingshi.

**Figure 2:**
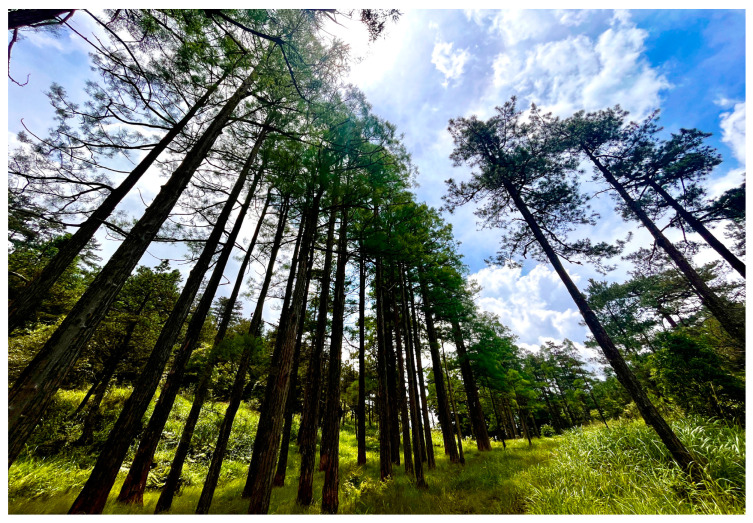
A *G. pensilis* population from Ningde (PNX).

The experimental materials were collected from 18 counties, across 7 cities in Fujian Province, comprising a total of 137 samples from 7 populations (Figure 1 and Figure 2; Table 1).

### 2.2. DNA Extraction and Sequencing

Fresh leaves were flash-frozen in liquid nitrogen and then transported back to the laboratory for storage in a −80 °C freezer. DNA was extracted using the rapid extraction kit (Beijing Biomed Gene Technology Co., Ltd., Beijing, China). The quality of the DNA was assessed using 1.5% agarose gel electrophoresis and Qbit quantification. Samples that met the quality and concentration requirements were packaged and sent to Wuhan Igenebook Biotechnology Co., Ltd. (Wuhan, China). (https://igenebook.biomart.cn/, accessed on 11 November 2024) for sequencing. The ddRAD library, with a length range of 300–500 bp was constructed for paired-end sequencing. The purified library was sequenced on the Illumina platform to obtain the sequences.

### 2.3. Acquisition of Single-Nucleotide Polymorphisms (SNPs) and Annotation

Based on the SacI sequence at the R1 end and the MseI as barcode information at the R2 end, the raw sequencing data were split accordingly. The split data were filtered using the default parameters of fastp v0.20.0 [18], and then, quality control was performed. This study conducted a RAD non-reference analysis using Stacks v2.65 [19]. The ustacks command was used, with the parameter M set to two to control the number of mismatches, and cstacks was used to build a catalog file containing the information of all loci, with the number of allowed mismatches between loci (n) set to two. After the catalog was constructed, the SNP mutation was compiled using the populations module v2.52 of the population software. To ensure the reliability of downstream analyses, these SNPs were further filtered using vcftools v0.1.16 [20], with the filtering criteria set to --max-missing 0.5 (maximum missing rate of 50%), --maf 0.05 (setting the minimum allele frequency to 0.05), and --min-meanDP 5 (ensuring a minimum genotype depth of five). After filtering, SNPs were used for subsequent analyses of population genetics.

### 2.4. Population Genetic Diversity Analysis

Using the populations module v2.52 within Stacks software, the inbreeding coefficient (*F_IS_*), observed heterozygosity (*H_o_*), and expected heterozygosity (*H_e_*) were calculated. Additionally, based on filtered SNP loci, genetic parameters, such as nucleotide diversity (π) and the genetic differentiation among populations (*F_st_*), were also computed.

### 2.5. Population Genetic Structure Analysis

The analysis was conducted using Admixture v1.3.0. First, the VCF file corresponding to the filtered SNPs was converted using Plink v1.90b4 [21]. Then, EIG v6.1.4 software was used for principal component analysis (PCA). CV error values were calculated for different *K* values, and the smallest CV error value was used to determine the best *K* value for the population.

### 2.6. Cluster Analysis

We used FastTree v2.1.11 software to construct the phylogenetic tree. The tree was built using the maximum likelihood (ML) method, based on the generalized time-reversible (GTR) model [22]. The constructed tree was visualized using the iTOL website (https://itol.embl.de/, accessed on 11 November 2024) [23].

## 3. Results

### 3.1. Sequencing Data

We employed ddRAD-seq to sequence 137 samples. The sequencing results underwent quality control, yielding a total of 18,024,931 clean reads. The average GC content was calculated to be 38.17%, with an average Q20 value of 97.65% and an average Q30 of 93.18%. These outcomes affirmed successful library construction and indicated that the sequencing data were of high quality, rendering them suitable for further analysis. In the analysis for variant detection, a de novo genomic analysis was performed using Stacks, resulting in an average fragment coverage of 8.47 across the 137 samples. Subsequently, by employing VCFtools v0.1.13 software for the filtration of SNP loci, out of 3,687,189 SNP loci, a total of 15,158 high-quality SNP loci were ultimately identified. Furthermore, these 15,158 high-quality SNP loci were subsequently employed for further analysis.

### 3.2. Genetic Diversity

The genetic diversity analysis of seven populations of *G. pensilis* indicated (Table 2) that the mean observed heterozygosity (*H_o_*) was 0.08630, the mean expected heterozygosity *(H_e_*) was 0.03475, and the mean nucleotide diversity (π) was 0.07239. Each population possessed unique alleles, with an average number of 320,590. Among these, the NingDe (ND) population harbored the most unique alleles (698,635), followed by FuZhou (FZ) (690,849), with SanMing (SM) having the least (57,671). The genetic diversity within populations was denoted by nucleotide diversity (π), which ranged from 0.06336 to 0.10104. The FZ population had the highest Pi value (π = 0.10104), while the SM population had the lowest values(π = 0.06336), which are indicative of higher and lower genetic diversity, respectively. The inbreeding coefficient (*F_IS_*) values ranged from 0.03746 to 0.23798, with an average of 0.11676. The ND population displayed the largest *F_IS_* value (*F_IS_* = 0.23798), while the SM population had the smallest value (*F_IS_* = 0.03746) (Table 2).

The genetic differentiation parameter (*F_st_*) values ranged from 0.126401 to 0.509101. The *F_st_* values between FZ and ND, NanPing (NP), SM, PuTian (PT), QuanZhou (QZ) and LongYan (LY), for PT with ND, NP, SM, and QZ, and for LY with ND, NP, SM, PT, and QZ, were greater than 0.25, suggesting substantial genetic differentiations. Additionally, the *F_st_* values between QZ and ND, NP and SM, and NP and SM ranged from 0.15 to 0.25, indicating significant genetic differentiations. Moreover, the *F_st_* values between ND and NP, SM ranged from 0.05 to 0.15, suggesting moderate genetic differentiations. It could be seen that the FZ population was the most genetically differentiated, with an average genetic differentiation parameter of 0.331261, compared to the other six populations (Table 3).

### 3.3. Genetic Structure

**Figure 3:**
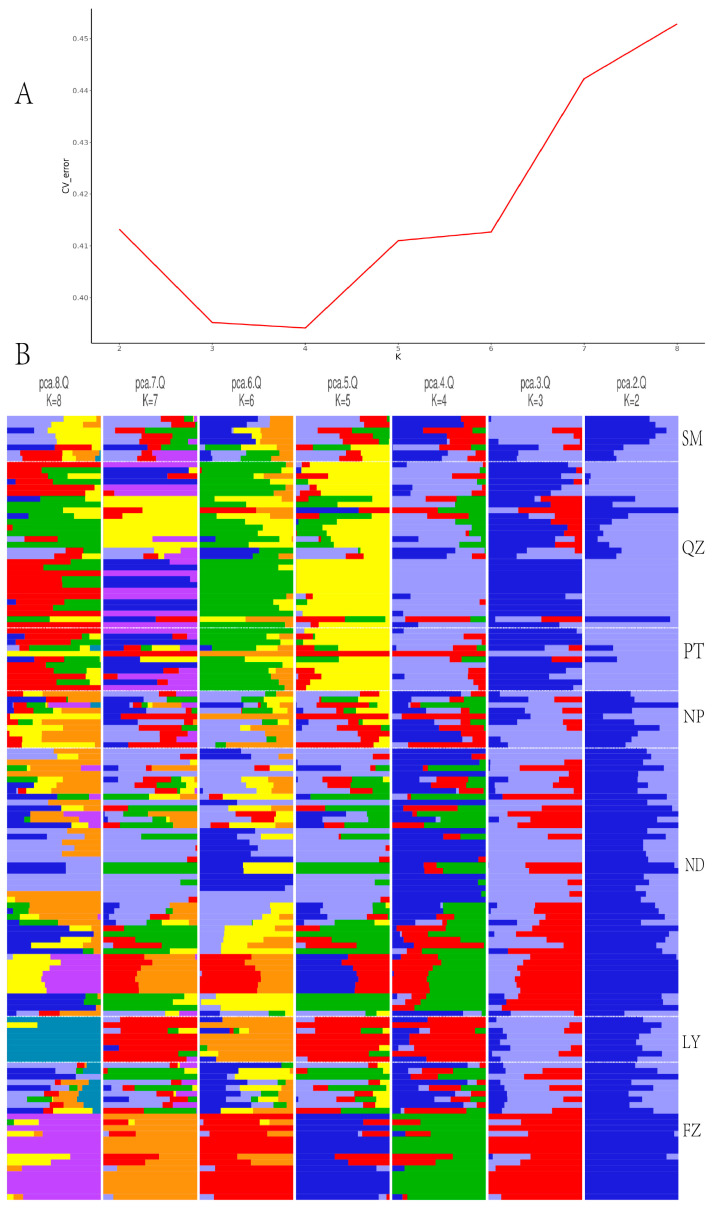
(**A**) Admixture validation error values corresponding to different *K* values. (**B**) Genetic structure of *G. pensilis* populations.

**Figure 4:**
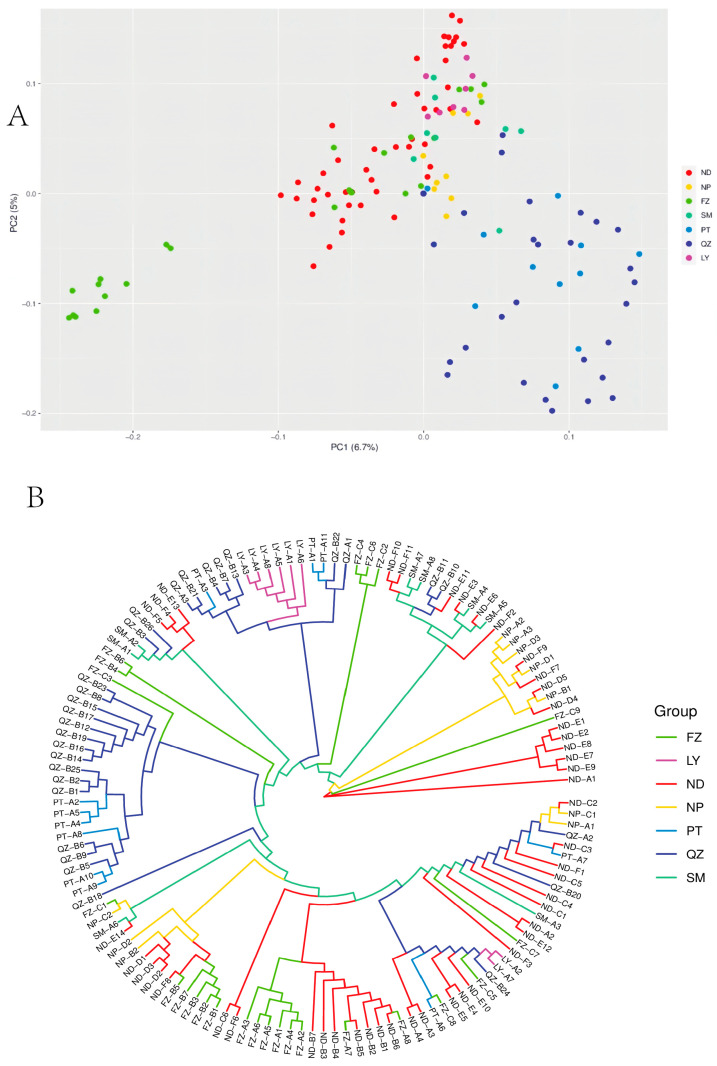
(**A**) The principal component analysis (PCA) of *G. pensilis* populations. The PCA plots for the 137 samples were created based on Principal Component 1 (horizontal axis) and Principal Component 2 (vertical axis). (**B**) Cluster analysis of 137 *G. pensilis* genotypes based on the approximate maximum likelihood method under the GTR model. ND: Ningde; NP: Nanping; FZ: Fuzhou; SM: Sanming; PT: Putian; QZ: Quanzhou; LY: Longyan.

Using Admixture software, a genetic structure analysis was conducted on the 15,158 SNP loci that were filtered. The range for the *K* values was set from two to eight, and the corresponding CV error values were obtained and are depicted in a line graph (Figure 3A). When *K* = 4, the CV error value was minimized, indicating that the 137 individuals could be divided into four distinct clusters (Figure 3B). According to the PCA, the individuals from QZ and PT were concentrated, while the individuals from ND, LY, FZ, SM and NP were also clustered together, with a few individuals from FZ scattered elsewhere (Figure 4A).

### 3.4. Cluster

Based on 15,158 SNPs, the 137 samples were clustered into eight branches (Figure 4B). The majority of individuals from FZ and ND, along with a few individuals from NP, were clustered together, indicating a more similar genetic composition and closer relationship. The vast majority of individuals from QZ and PT were also clustered on the same branch, highlighting the close relationship and geographical proximity. The majority of individuals from LY were clustered with those from QZ and PT. The results from the tree suggest that the 137 individuals were geographically clustered into two major genetic branches. The four populations from ND, FZ, NP, and SM are located in the northern part of Fujian Province, whereas those from LY, QZ, and PT are located in the southern part.

## 4. Discussion

Previous studies employing simple sequence repeats (SSR) and ISSR molecular markers to detect the genetic diversity of *G. pensilis* have found these methods are not only time-consuming and labor-intensive but also costly [24,25]. Currently, the development of SNP markers based on reduced-representation genome sequencing has become mainstream [26,27,28,29]. We utilized this approach for sequencing 137 *G. pensilis* individuals, achieving read recovery rates of over 94% for each sample, with an average of 98.21%, an average GC content of 38.17%, a Q20 mean of 97.65%, and a Q30 mean of 93.18%. The low GC content indicated well-constructed libraries, and high Q30 values indicated low error rates in bases, ensuring high-quality base data and reliable sequencing results. The study acquired a total of 3,687,189 SNP loci by setting a filtering criterion with a minimum genotype depth > 5, a sample missing rate < 0.5, and an allele frequency > 0.05, eventually obtaining 15,158 high-quality SNP loci. The results demonstrate the valuable application and promising future of dd-RAD in the study of genetic variation in *G. pensilis*.

The genetic diversity of a species reveals its potential to adapt to environmental changes and provides an effective assessment of species survival, especially for endangered species. Assessing genetic variance is a crucial method for conserving endangered species resources [30]. Genetic parameters such as nucleotide diversity (π), observed heterozygosity (*H_o_*), and expected heterozygosity (*H_e_*) can indicate the level of population genetic diversity. Nucleotide diversity is a comprehensive indicator for assessing species population genetic diversity [19]. The seven populations using SNP molecular markers indicated low genetic diversity (π = 0.07239, *H_o_* = 0.08630, *H_e_* = 0.03475). Similar results were obtained by Tam et al. [31] using chloroplast microsatellite markers (cpSSRs) for 134 *G. pensilis* individuals (*H_o_* = 0.076, *H_e_* = 0.087). Compared to other tree species, a study of 233 fir individuals using reduced-representation genome sequencing found medium genetic diversity (*H_o_* = 0.215, *H_e_* = 0.233) [32], and a genetic analysis of 13 populations from the genus *Cryptomeria* yielded similar levels (*H_o_* = 0.2170, *H_e_* = 0.2030) [33]. A study on the genetic diversity and structure of seven ancient *Cryptomeria japonica* var. Sinensis populations also reported comparable results (*H_o_* = 0.143) [34]. Therefore, it can be seen that *G. pensilis* has lower genetic diversity compared to other tree species or congeneric species. The inbreeding coefficient (*F_IS_*), which measures the presence of heterozygotes in a population, ranged from 0.03746 to 0.23798 across the seven *G. pensilis* populations studied, with a mean of 0.11676, indicating possible inbreeding depression. The low genetic diversity was identified as a primary factor contributing to the endangerment of *G. pensilis*. If the observed heterozygosity is higher than the expected heterozygosity, it may indicate that there was gene exchange between two previously isolated populations. Most populations have high *F_st_*, indicating significant genetic differentiation between populations, which may be due to geographic isolation, ecological factors, or human activities. In the future, on the basis of genome sequencing, resequencing will reveal the genetic variation of *G. pensilis* populations more accurately.

In Pingnan County (PNX) and Youxi County (YXX), there were fewer individuals with DBH between 80 and 127 cm, and the corresponding ages ranged from 192 to 357 years [4]. Obtaining a population’s genetic structure provides the theoretical basis for the conservation of *G. pensilis* resources. Based on the PCA findings, the 137 samples were primarily concentrated in two geographic distributions across the south–north direction of Fujian Province. This aligned with the tree results, which showed QZ and PT forming a major genetic branch, with another branch composed mostly of FZ, ND, NP, and other populations. The *F_ST_* values, ranging between 0 and 1, indicated the degree of genetic differentiation between populations, with values greater than 0.25 suggesting significant differentiation [35]. In this study, among the 21 pairwise *F_ST_* values from seven groups, 15 *F_ST_* values were greater than 0.25. This indicated significant genetic differentiation between the populations of FZ and ND, NP, SM, PT, QZ, and LY, substantial genetic differentiation between PT and ND, NP, and SM, and a high level of genetic differentiation between LY and ND, NP, SM, PT, and QZ. The results show that the degree of genetic differentiation within *G. pensilis* is high (*F_ST_* = 0.39558). Using an ISSR molecular marker, Wu et al. [36] analyzed the genetic diversity of 112 individuals from eight *G. pensilis* populations and found a *G_ST_* value of 0.2982. This value is lower than the average ISSR genetic differentiation coefficient (*G_ST_* value = 0.34) reported by Nybom et al. [37], indicating a substantial degree of genetic differentiation among *G. pensilis* populations, which is consistent with the findings of our study.

The stability and evolutionary potential of species depend on their genetic diversity, and the ecological and economic value of species also depends on their excellent genetic composition, so the ultimate goal of species conservation is to conserve their genetic diversity. Understanding the distribution pattern of genetic structure is the theoretical basis for people to make rational decisions [38]. The genetic diversity of a population is the sum of the genetic variation of different individuals within the same population. Some individuals of a species can adapt to environmental variations and fix the variant gene, and then survive in a specific environment. After several generations of the surviving individuals reproducing, more individuals in the population will carry this gene, and then, the genetic diversity of the population will be higher compared to that of previous generations, with the ability to adapt to the environment also increasing. This indicates that genetic diversity plays an important role in the survival and adaptability of species [39]. The characteristics of low genetic diversity and high population genetic differentiation in *G. pensilis* are correlated with their geographical environment. After historical climatic changes, the distribution area of *G. pensilis* has drastically reduced, and its strict environmental requirements have led to a rapid decline in numbers and the subsequent extinction of several populations. As populations decreased quickly in size and number, their heterozygosity dropped, causing a decrease in genetic diversity levels, which are lower compared to other tree species [40]. Due to the scattered distribution of *G. pensilis* and human activities, clear habitat isolation has occurred, and as population sizes have diminished, gene flow between populations has increasingly reduced, further exacerbating its decline in genetic diversity [41]. One of the core principles of conservation biology is the study of the endangerment mechanisms of endangered species. The use of reduced-representation genome sequencing to study the population genetic diversity of *G. pensilis* is significant for exploring its endangerment mechanisms and effectively protecting wild populations.

Given the endangered status of *G. pensilis*, it is recommended to strengthen the protection of its native habitats [42], especially conserving populations with high genetic diversity, such as the FZ population, and paying attention to the protection of the ecosystems surrounding populations by establishing nature reserves to maximize the protection of their higher genetic diversity. Most *G. pensilis* populations are distributed in a scattered fashion, in small numbers, and the seedlings have low survival rates and strict habitat requirements, making natural reproduction difficult. Translocation conservation could effectively enhance the gene flow between populations, serving as one of the essential methods for protecting endangered tree species [43]. By introducing specimens from various locations, the genetic resource exchange of *G. pensilis* can be increased, and investigating suitable environments for their growth can ensure that it has favorable conditions for growth elsewhere, enabling successful natural regeneration. Since the 1970s, Southern China has sequentially initiated the artificial cultivation of *G. pensilis* by selectively breeding certain genotypes. However, this has led to a deficiency in genetic diversity due to inbreeding. Therefore, it is recommended to concurrently undertake artificial cultivation and natural population protection to maximize the genetic diversity, significantly contributing to the conservation and restoration efforts of the *G. pensilis* population [44]. Developing gene resource conservation strategies based on population structure is crucial for maintaining the future genetic diversity and sustainability of the *G. pensilis* populations.

The study on *G. pensilis* is constrained by its limited natural regeneration capacity and severely degraded habitat, which resulted in a significant population decline. Additionally, challenges such as the absence of a reference genome and low genetic diversity further complicate conservation efforts. Future endeavors will acquire genome and use resequencing to elevate the conservation genetics of *G. pensilis*, with the objective of elucidating its phylogeography and genetic structure, thereby reinforcing conservation strategies. During the artificial population expansion of *G. pensilis*, it is crucial to introduce and cultivate populations with distinct genetic differences from diverse provenances to enhance heterogeneity and increase genetic variation, maximizing the preservation of genetic diversity. The establishment of a germplasm resource bank for *G. pensilis* will promote genetic exchange among reproductive materials from different provenances, offering high-quality and diverse genetic resources for population restoration and ensuring the effective conservation of this species.

## 5. Conclusions

We have unearthed 15,158 high-quality SNPs in *G. pensilis*, which reveal a state of diminished genetic diversity and elevated genetic differentiation among populations. The population structure analysis suggests partitioning into four distinct clusters exhibiting substantial gene flow, while the principal component analysis (PCA) corroborates a geographical demarcation along the north–south axes. The precarious status of *G. pensilis* can be attributed to a confluence of factors, including suboptimal natural regeneration capabilities, anthropogenic disturbances, and the impacts of climatic variations.

## Figures and Tables

**Table 1:** Sample details of *Glyptostrobus pensilis*.

Population	Sampling Location	Longitude (E)	Latitude (N)	Number of Samples	Total Population
Ningde (ND)	Xiapuxian (XPX)	119°57′	27°03′	4	47
	Fu’anshi (FAS)	119°27′	27°10′	7	
	Zhouningxian (ZNX)	119°14′	27°08′	6	
	Jiaochengqu (JCQ)	119°23′	26°43′	5	
	Pingnanxian (PNX)	118°58′	26°58′	14	
	Gutianxian (GTX)	119°10′	26°35′	11	
Nanping (NP)	Puchengxian (PCX)	118°28′	27°41′	3	10
	Zhenghexian (ZHX)	119°10′	27°13′	2	
	Jian’oushi (JOS)	118°28′	27°02′	2	
	Shaowushi (SWS)	117°26′	27°31′	3	
Fuzhou (FZ)	Luoyuanxian (LYX)	119°20′	26°29′	8	24
	Minhouxian (MHX)	119°04′	26°18′	7	
	Yongtaixian (YTX)	118°46′	25°54′	9	
Sanming (SM)	Youxixian (YXX)	118°19′	26°04′	8	8
Putian (PT)	Xianyouxian (XYX)	118°48′	25°30′	11	11
Quanzhou (QZ)	Dehuaxian (DHX)	118°16′	25°31′	3	29
	Yongchunxian (YCX)	118°18′	25°25′	26	
Longyan (LY)	Zhangpingshi (ZPS)	117°14′	25°03′	8	8

**Table 2:** Genetic diversity for seven populations of *G. pensilis*.

Population	Ap	*H_o_*	*H_e_*	π	*F_IS_*
ND	698635	0.07540	0.03919	0.06638	0.23798
NP	60156	0.08858	0.02815	0.06696	0.04171
FZ	690849	0.10084	0.04663	0.10104	0.18629
SM	57671	0.08288	0.02627	0.06336	0.03746
PT	134862	0.08124	0.03046	0.06396	0.07501
QZ	411223	0.07708	0.04035	0.07578	0.16429
LY	190731	0.09805	0.03217	0.06925	0.07455
Average	320590	0.08630	0.03475	0.07239	0.11676

ND: Ningde; NP: Nanping; FZ: Fuzhou; SM: Sanming; PT: Putian; QZ: Quanzhou; LY: Longyan.; Ap: alleles; *H_o_*: observed heterozygosity; *H_e_*: expected heterozygosity; π: nucleotide diversity; *F_IS_*: inbreeding coefficient.

**Table 3:** *F_ST_* value for each population of *G. pensilis*.

	ND	NP	FZ	SM	PT	QZ	LY
ND		0.129501	0.273062	0.126401	0.278293	0.216825	0.281516
NP			0.283058	0.245755	0.498077	0.231838	0.429099
FZ				0.347162	0.432882	0.343870	0.307534
SM					0.473856	0.215913	0.469676
PT						0.367361	0.509101
QZ							0.395584

ND: Ningde; NP: Nanping; FZ: Fuzhou; SM: Sanming; PT: Putian; QZ: Quanzhou; LY: Longyan.

## Data Availability

The ddRAD-seq data are available in the Genome Sequence Archive (GSA) of the NGDC (National Genomics Data Center) under accession number PRJCA28558 (CRA018703).

## References

[1] Averyanov L.V., Nguyen T.H., Sinh K.N., Pham T.V., Lamxay V., Bounphanmy S., Lorphengsy S., Phan L.K., Lanorsavanh S., Chantthavongsa K. (2014). Gymnosperms of Laos. Nord. J. Bot..

[2] Chen Y.Q., Wang R.J., Zhu S.S., Jiang A.L., Zhou L.X. (2016). Population status and conservation strategy of the rare and endangered plant *Glyptostrobus pensilis* in Guangzhou. Trop. Geogr.

[3] Ye X., Zhang M., Yang Q., Ye L., Liu Y., Zhang G., Chen S., Lai W., Wen G., Zheng S. (2022). Prediction of suitable distribution of a critically endangered plant *Glyptostrobus pensilis*. Forests.

[4] Tang C.Q., Yang Y., Momohara A., Wang H.C., Luu H.T., Li S., Song K., Qian S., LePage B., Dong Y.F. (2019). Forest characteristics and population structure of *Glyptostrobus pensilis*, a globally endangered relict species of southeastern China. Plant Divers..

[5] Pueyo-Herrera P., Tang C.Q., Matsui T., Ohashi H., Qian S., Yang Y., Herrando-Moraira S., Nualart N., López-Pujol J. (2023). Ecological niche modeling applied to the conservation of the East Asian relict endemism *Glyptostrobus pensilis* (Cupressaceae). New For..

[6] Frankham R., Briscoe D.A., Ballou J.D. (2002). Introduction to Conservation Genetics.

[7] LePage B.A. (2007). The taxonomy and biogeographic history of *Glyptostrobus* Endlicher (Cupressaceae). Bull. Peabody Mus. Nat. Hist..

[8] Li F., Xia N. (2005). Population structure and genetic diversity of an endangered species, *Glyptostrobus pensilis* (Cupressaceae). Bot. Bull. Acad. Sin..

[9] Wu Z.Y., Liu J.F., Hong W., Pan D.M., Zheng S.Q., He Z.S. (2012). Genetic diversity of different life stage population of *Glyptostrobus pensilis*, an endangered plant in China: ISSR analysis. Chin. J. Ecol..

[10] Le S., Van Le T. (2024). Genetic diversity and population structure of natural provenances of *Sonneratia caseolaris* in Vietnam. J. Genet. Eng. Biotechnol..

[11] Umesh Kanna S., Parthiban K.T., Senthilraja K., Venkatesan S., Udhaya Nandhini D., Mohan Kumar S., Dhasarathan M., Kumaresan P., Jaswanth Sai M., Raveendran M. (2024). Genetic Diversity and Structure of *Terminalia bellerica* (Gaertn. Roxb.) Population in India as Revealed by Genetic Analysis. Plants.

[12] Danusevičius D., Rajora O.P., Kavaliauskas D., Baliuckas V., Augustaitis A. (2024). Stronger genetic differentiation among within-population genetic groups than among populations in Scots pine provides new insights into within-population genetic structuring. Sci. Rep..

[13] Szyp-Borowska I., Zawadzka A., Wojda T., Klisz M. (2023). Analysis of the genetic diversity and population structures of black locust (L.) stands in Poland based on simple sequence repeat markers. Folia For. Pol..

[14] Hodel R.G., Chen S., Payton A.C., McDaniel S.F., Soltis P., Soltis D.E. (2017). Adding loci improves phylogeographic resolution in red mangroves despite increased missing data: Comparing microsatellites and RAD-Seq and investigating loci filtering. Sci. Rep..

[15] Zhu Z., Sun B., Lei J. (2021). Specific-locus amplified fragment sequencing (SLAF-Seq) as high-throughput SNP genotyping methods. Crop Breeding. Methods in Molecular Biology.

[16] Peterson B.K., Weber J.N., Kay E.H., Fisher H.S., Hoekstra H.E. (2012). Double digest RADseq: An inexpensive method for de novo SNP discovery and genotyping in model and non-model species. PLoS ONE.

[17] Baird N.A., Etter P.D., Atwood T.S., Currey M.C., Shiver A.L., Lewis Z.A., Selker E.U., Cresko W.A., Johnson E.A. (2008). Rapid SNP discovery and genetic mapping using sequenced RAD markers. PLoS ONE.

[18] Chen S.F., Zhou Y.Q., Chen Y.R., Gu J. (2018). Fastp: An Ultra-Fast All-in-One FASTQ Preprocessor. Bioinformatics.

[19] Catchen J., Hohenlohe P.A., Bassham S., Amores A., Cresko W.A. (2013). Stacks: An analysis tool set for population genomics. Mol. Ecol..

[20] Danecek P., Auton A., Abecasis G., Albers C.A., Banks E., DePristo M.A., Handsaker R.E., Lunter G., Marth G.T., Sherry S.T. (2011). The variant call format and VCFtools. Bioinformatics.

[21] Purcell S., Neale B., Todd-Brown K., Thomas L., Ferreira M.A., Bender D., Maller J., Sklar P., de Bakker P.I., Daly M.J. (2007). PLINK: A tool set for whole-genome association and population-based linkage analyses. Am. J. Hum. Genet.

[22] Price M.N., Dehal P.S., Arkin A.P. (2009). FastTree: Computing large minimum evolution trees with profiles instead of a distance matrix. Mol. Biol. Evol..

[23] Letunic I., Bork P. (2024). Interactive Tree of Life (iTOL) v6: Recent updates to the phylogenetic tree display and annotation tool. Nucleic Acids Res..

[24] Li Y., Lin X.Y., Ruhsam M., Chen L., Wu X.T., Wang M.Q., Thomas P.I., Wen Y.F. (2019). Development of chloroplast microsatellite markers for the critically endangered conifer *Glyptostrobus pensilis* (Cupressaceae) using transcriptome data. Silvae Genet..

[25] Wu X., Ruhsam M., Wen Y., Thomas P.I., Worth J.R., Lin X., Wang M., Li X., Chen L., Lamxay V. (2020). The last primary forests of the Tertiary relict *Glyptostrobus pensilis* contain the highest genetic diversity. For. Int. J. For. Res..

[26] Ogden R., Gharbi K., Mugue N., Martinsohn J., Senn H., Davey J.W., Pourkazemi M., McEwing R., Eland C., Vidotto M. (2013). Sturgeon conservation genomics: SNP discovery and validation using RAD sequencing. Mol. Ecol..

[27] Pan Y., Wang X., Sun G., Li F., Gong X. (2016). Application of RAD sequencing for evaluating the genetic diversity of domesticated *Panax notoginseng* (Araliaceae). PLoS ONE.

[28] Miller M.R., Atwood T.S., Eames B.F., Eberhart J.K., Yan Y.L., Postlethwait J.H., Johnson E.A. (2007). RAD marker microarrays enable rapid mapping of zebrafish mutations. Genome Biol..

[29] Longo A., Kurta K., Vanhala T., Jeuthe H., de Koning D.J., Palaiokostas C. (2024). Genetic diversity patterns in farmed rainbow trout (*Oncorhynchus mykiss*) populations using genome-wide SNP and haplotype data. Anim. Genet..

[30] Mable B.K. (2019). Conservation of adaptive potential and functional diversity: Integrating old and new approaches. Conserv. Genet..

[31] Tam N.M., Duy V.D., Xuan B.T.T., Duc N.M. (2013). Genetic variation and population structure in Chinese water pine (*Glyptostrobus pensilis*): A threatened species. Indian J. Biotechnol..

[32] Jing Y., Bian L., Zhang X., Zhao B., Zheng R., Su S., Ye D., Zheng X., El-Kassaby Y., Shi J. (2023). Genetic diversity and structure of the 4th cycle breeding population of Chinese fir (*Cunninghamia lanceolata* (lamb.) hook). Front. Plant Sci..

[33] Li X., Cai M., Wang M., Wu X., Ueno S., Uchiyama K., Onuma Y., Dai M., Tao Y., Wen Y. (2024). Genetic diversity, genetic differentiation and demographic history of *Cryptomeria* (Cupressaceae), a tertiary relict plant in East Asia based on RAD sequencing. Eur. J. For. Res..

[34] Cai M., Wen Y., Uchiyama K., Onuma Y., Tsumura Y. (2020). Population genetic diversity and structure of ancient tree populations of *Cryptomeria japonica* var. sinensis based on RAD-seq data. Forests.

[35] Wright S. (1984). Evolution and the Genetics of Populations, Volume 4: Variability Within and Among Natural Populations.

[36] Wu Z.Y., Liu J.F., Zhang X.P., Hong W. (2010). Genetic Diversity of the Mesozoic Relict Plant Metasequoia Populations Revealed by ISSR Analysis. J. Chin. Bot. Gard..

[37] Nybom H., Bartish I.V. (2000). Effects of life history traits and sampling strategies on genetic diversity estimates obtained with RAPD markers in plants. Perspect. Plant Ecol. Evol. Syst..

[38] Agarwal M., Shrivastava N., Padh H. (2008). Advances in molecular marker techniques and their applications in plant sciences. Plant Cell Rep..

[39] Frankham R. (2005). Genetics and extinction. Biol. Conserv..

[40] Zhang J., Fischer G.A. (2021). Reconsideration of the native range of the Chinese Swamp Cypress (*Glyptostrobus pensilis*) based on new insights from historic, remnant and planted populations. Glob. Ecol. Conserv..

[41] Cheng Z., Weng C., Steinke S., Mohtadi M. (2018). Anthropogenic modification of vegetated landscapes in southern China from 6000 years ago. Nat. Geosci..

[42] Yu J., Wang C., Wan J., Han S., Wang Q., Nie S. (2014). A model-based method to evaluate the ability of nature reserves to protect endangered tree species in the context of climate change. For. Ecol. Manag..

[43] Pritchard D.J., Fa J.E., Oldfield S., Harrop S.R. (2012). Bring the captive closer to the wild: Redefining the role of ex situ conservation. Oryx.

[44] Zhang Y.T., Yang J.J., Guo Z.H., Mo J.X., Cui J.B., Hu H.L., Xu J. (2020). Comparative analyses and phylogenetic relationships between *Cryptomeria fortunei* and related species based on complete chloroplast genomes. Phyton-Int. J. Exp. Bot..

